# Inefficacy of N-acetylcysteine in mitigating cue-induced amphetamine-seeking

**DOI:** 10.1016/j.addicn.2023.100119

**Published:** 2023-07-14

**Authors:** Troy D. Fort, Mary E. Cain

**Affiliations:** Kansas State University, United States

**Keywords:** Astrocytes, Incubation of craving, Glutamate homeostasis, Relapse, Amphetamine

## Abstract

Glutamatergic imbalances are characteristic of SUDs. Astrocytic and neuronal transporters help regulate glutamate homeostasis and disruptions in this homeostasis engender SUD. The cysteine-glutamate exchanger (xCT) is primarily localized on astrocytes and maintains glutamate concentrations. This process is disrupted by cocaine use, and the therapeutic *N*-acetylcysteine (NAC) lowers cue-induced relapse to cocaine by restoring xCT function. However, little research has shown how these effects extend to other psychostimulants, such as amphetamine (AMP). Here, we assessed xCT expression following relapse to AMP cues, and if NAC can attenuate relapse via changes to astrocyte and xCT expression. We administered NAC (100 mg/kg ip) daily during a 14-day abstinence period following AMP (0.1 mg/kg/infusion; 2 h sessions) self-administration. Relapse was tested following one (WD 1) or 14 days (WD 14) of withdrawal. The overall number of astrocytes was also quantified within the medial prefrontal cortex (mPFC) and nucleus accumbens (ACb). NAC failed to lower cue-induced AMP craving via cue-induced relapse and reinstatement testing. Cue-induced craving did not increase from WD 1 to WD 14. AMP-exposed rats had greater astrocyte counts in the mPFC and ACb when compared AMP-naïve rats. Repeated injection with NAC decreased xCT expression within the mPFC and ACb. Overall, these results suggest that NAC may be an ineffective treatment option for lowering cue-induced relapse to AMP. Further, the results suggest that stimulating xCT via NAC may not be an effective therapeutic approach for decreasing cue-seeking for AMP.

## 1. Introduction

Psychostimulant overdoses, such as amphetamine, have increased by 22.3% annually from 2008 to 2017 [1]. This surge in amphetamine abuse is especially prevalent in college-aged individuals [2]. Importantly, this population is of significant concern due to the ongoing and dynamic changes in synaptic pruning and neuroplasticity that occur during late adolescence [3]. Recent data have shown that amongst college students, psychostimulants constitute 73% of all non-medical drug use. Of those individuals, 81.2% reported non-prescription Adderall use, thus making dextroamphetamine the most commonly abused psychostimulant in this age demographic [2]. Therefore, it is critical for us to characterize the biological mechanisms that perpetuate amphetamine-use disorder.

Addiction is a chronic disorder characterized by repeated episodes of abstinence and relapse. The drug-craving that ultimately leads to relapse remains a key obstacle in treatments designed to attenuate relapse and SUDs. Drug craving is often induced through drug-associated cues which increase motivation to obtain drug-associated outcomes, referred to here as cue-induced craving. Even after long periods of drug abstinence, drug-associated cues are still able to induce relapse, thus perpetuating addiction. Further exacerbating this issue is the finding that cue-induced drug craving increases concurrently with drug abstinence. There is a broad wealth of literature indicating that cue-induced craving increases linearly with the duration of drug abstinence. This relationship between the increase in the motivational impact of drug-associated cues and drug abstinence is referred to as *incubation of drug craving* [4–6].

The glutamate homeostasis theory of addiction suggests that addiction and cue-induced craving occurs via disruption in the proper release and elimination of glutamate in the synaptic and extrasynaptic spaces [7]. These disruptions in glutamate homeostasis are particularly notable within the nucleus accumbens (ACb), a key region within the mesocorticolimbic pathway. Maintenance of glutamate homeostasis within this pathway involves a number of astrocytic and neuronal glutamate transporters. Astroglial glutamate transporters and receptors work in concert to maintain the optimal level of glutamate in the synaptic and extrasynaptic spaces. One such transporter, the cysteine-glutamate exchanger (xCT), is responsible for exporting glutamate into the extrasynaptic space by exchanging one intracellular glutamate molecule for one extracellular cysteine molecule and is predominately expressed on the surface of astrocytes [7,8]. Within the ACb, approximately 60% of all extracellular glutamate is derived from cystine-glutamate exchange [8]. Additionally, the excitatory amino acid transporter, GLT1, is responsible for preventing ~90% of glutamate spillover, from the synaptic to extrasynaptic space [9]. GLT1 is primarily localized in the synaptic cleft and is responsible for trafficking extracellular glutamate back to the astrocyte to await export during cysteine-glutamate exchange [10].

Both xCT and GLT1 work to maintain optimal glutamatergic tone on perisynaptic metabotropic receptors (mGluRs), in particular the mGluR2/3 autoreceptors. The mGluR2/3 autoreceptors are primarily localized on presynaptic neurons and are responsible for regulating synaptic glutamate release from the presynaptic neuron. When glutamate is available to bind mGluR2/3, via export of xCT on the nearby astrocyte, presynaptic glutamate release is halted. As such, optimal glutamatergic tone on mGluR2/3 engenders homeostatic levels of synaptic and extrasynaptic glutamate.

Cocaine has been shown to downregulate the expression of xCT and GLT1 in the mPFC and ACb [11–13]. This downregulation leads to a decrease in glutamatergic tone on mGluR2/3 and concomitant disruptions in glutamate homeostasis. While downregulations in xCT and GLT1 are reasonably well characterized in self-administration models of cocaine [6], these impacts resulting from exposure to other psychostimulants and subsequent glutamate homeostasis are significantly less characterized. The effect that methamphetamine has on xCT and GLT1 expression is largely dose-dependent, with subtoxic doses failing to decrease either xCT [14] or GLT1 expression [15]. Overall, these results show a potential departure in how glutamate homeostasis may be disrupted by different psychostimulants. It is one goal of the current experiment to explore how these glial systems, particularly xCT, may be disrupted in response to amphetamine. This knowledge will help to inform the most logical and efficacious targets for therapeutic interventions to treat cue-induced relapse to amphetamine.

Therapeutics designed to restore glutamate homeostasis and attenuate associated drug-seeking have shown potential therapeutic efficacy. One such therapeutic, the cysteine prodrug *N*-acetylcysteine (NAC), has shown promise in mitigating cue-induced craving in both preclinical models of SUD and individuals suffering from SUD [16]. NAC has been shown to attenuate decreases in extracellular glutamate that occur within the ACb core during cocaine withdrawal [11] through several mechanisms. The mechanism of action underscoring NAC’s potential efficacy lies in its ability to restore GLT1 expression within the ACb [16]. In addition, NAC restores cysteine-glutamate exchange via xCT [17]. NAC supplies extracellular cysteine to stimulate xCT-mediated glutamate release. This release of extracellular cysteine via xCT is sufficient to restore glutamate homeostasis [11]. This stimulation of the xCT system increases extrasynaptic glutamate levels sufficient to bind mGluR2/3, thus inhibiting presynaptic glutamate release and concomitant neuronal potentiation [18,19]. Indeed, inhibition of xCT-mediated extracellular glutamate release precludes NAC-induced normalization of extracellular glutamate levels within the ACb core, thus suggesting that NAC-induced normalization of glutamate homeostasis is heavily reliant on xCT-mediated glutamate release [11].

While the majority of research has suggested NAC’s efficacy in treating cue-induced relapse to cocaine [16,20], NAC has shown potential efficacy in reducing cue-induced craving for methamphetamine [21]. Importantly, though Siemsen et al. [21] found that NAC was effective in attenuating cue-induced reinstatement for methamphetamine, they did not find that methamphetamine exposure during self-administration produced downregulations in GLT1. In summary, NAC exhibits several mechanisms of action that help demonstrate its potential utility in attenuating several aspects of glutamatergic imbalance, and most notably, in helping to mitigate cue-induced drug-seeking. However, its efficacy in attenuating cue-induced relapse and reinstatement to amphetamine has not been examined.

In the current study, we aimed to extend these findings of NAC’s potential efficacy in attenuating cue-induced craving to cocaine and methamphetamine to amphetamine relapse. In Experiment 1, we evaluated NAC’s efficacy in lowering cue-induced relapse to amphetamine following acute and protracted abstinence to amphetamine. We then assessed the expression of xCT and GFAP, an intermediate filament-III protein that is localized in astrocytes in the central nervous system [7, 8,22]. In Experiment 2, we evaluated whether the efficacy of NAC was dependent on the presence of extinction training. To do so, we gave NAC during daily extinction sessions following amphetamine self-administration and then assessed cue-induced reinstatement on Withdrawal Day 14.

## 2. Methods and materials

### 2.1. Animal husbandry

One-hundred and one male Sprague-Dawley rats were obtained from Charles River Laboratories. All rats weighed approximately 250 g when arriving at Kansas State University. All animals were pair-housed in plastic shoebox cage containing bedding and wire tops. All animals were handled weekly during scheduled cage changes. All experimental procedures were conducted in accordance with the Institutional Animal Care and Use Committee at Kansas State University and the NIH guidelines for the care and use of laboratory animals.

### 2.2. Apparatus

All training and testing was conducted in standard operant chambers (Med Associates). The standard operant chambers are enclosed within ventilated sound-attenuating chambers. Each chamber contained two levers, house light, a tone generator, a tether (to administer amphetamine; chemical-resistant tubing covered by stainless steel extension spring; the spring prevents chewing of the tubing), lights above the lever, self-administration pump, and liquid presentation magazine (dipper 0.1 ml). The opening for the magazine is 5 × 4.2 cm and this allows the rat to head poke to drink from the dipper during lever press training.

### 2.3. Procedures

#### 2.3.1. Lever-press training

**Fig. 1.**
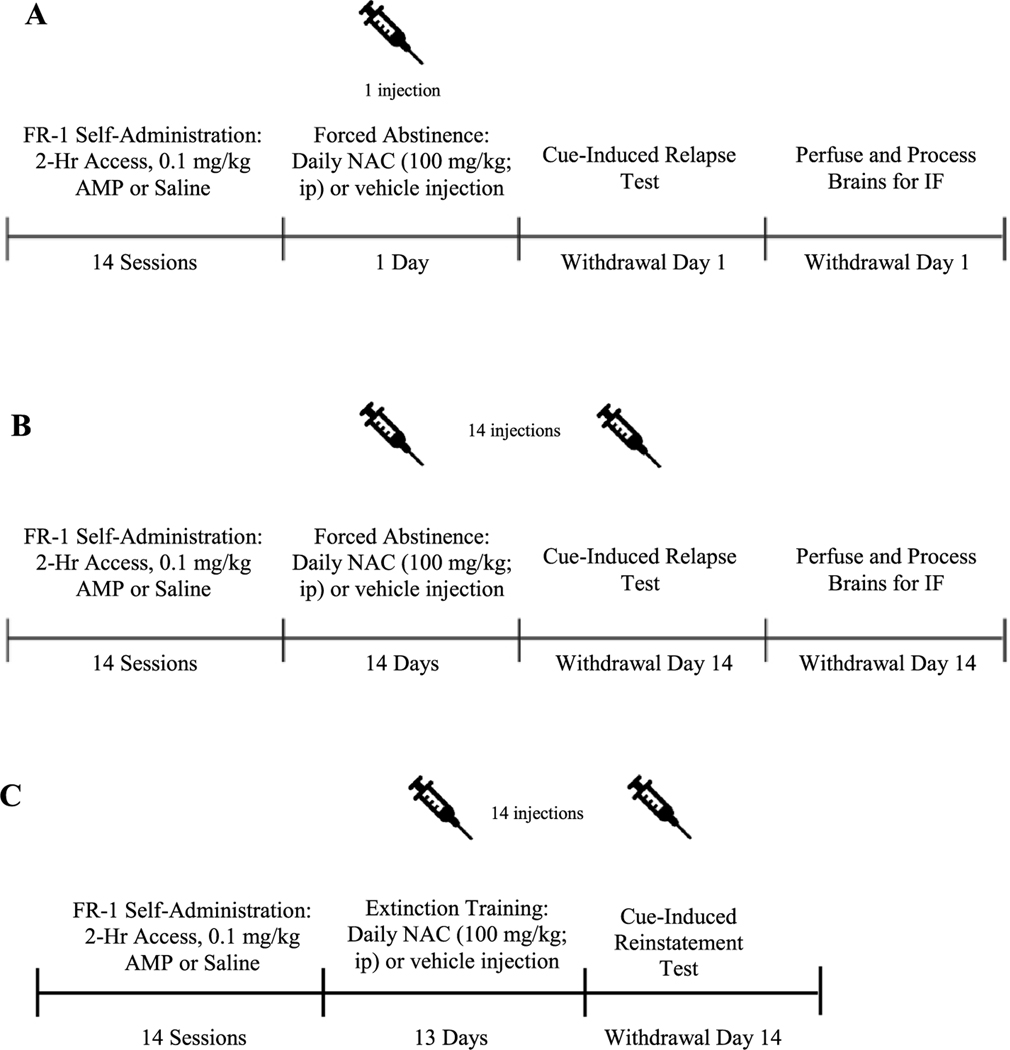
Experimental timeline. (A) Timeline for animals tested under acute withdrawal on Withdrawal Day 1. (B) Timeline for animals tested under protracted withdrawal on Withdrawal Day 14. (C) Timeline for animals undergoing extinction training and subsequent cue-induced reinstatement testing on Withdrawal Day 14.

A figure showing a general experimental timeline can be referenced in Fig. 1. After habituating to the colony for at least a week, all animals underwent several operant training sessions to facilitate the acquisition of the lever-reinforcer association. To increase motivation for water reinforcement, all animals were water-deprived for 12–18 h prior to each training session. This water deprivation duration has been used in previous literature for lever press training [23–26]. The reinforcer for all lever press training sessions was a presentation of 0.1 ml of water. During the first session, water access was not contingent on lever pressing. During the second training session, only one lever was presented to the animal and water reinforcement was contingent on active lever pressing. Following successful acquisition of the lever-reinforcement contingency, all animals underwent three more fixed-ratio 1 (FR-1) water self-administration sessions. During the three FR-1 water training sessions, all animals were presented with both the active lever, which resulted in water reinforcement, and an inactive lever which had no programmed consequence. After completion of each session, rats were returned to their home cages and were given ad libitum access. We did not observe weight loss or signs of dehydration in the rats during the week of water restriction and all rats readily learned to lever press.

#### 2.3.2. Surgical procedures

Following acquisition of lever pressing, all animals were given 3–4 days of post-training recovery. Rats were deeply anesthetized with isoflurane (~ 5%) and implanted with indwelling jugular catheters using methods described previously [27,28]. Polyurethane catheters (12 cm in length, 0.2 mm internal diameter: SAI) were inserted through a dorsal incision in the animal’s back, tunneled under the skin and into the animal’s left jugular vein. Catheter tubing was subcutaneously connected to a 22-gage back-mounted cannula (Plastics One: Roanoke, VA) and sutured to surgical mesh (Biomedical Structures; Warwick, RI). To prevent animals from chewing or damaging back mounts, a stainless-steel bolt was threaded onto the end of the back mount. During the first 2 days of recovery rats were treated with Meloxicam (2.0 mg/kg after surgery;1.0 mg/kg thereafter) in order to alleviate pain. Baytril^®^ (Enrofloxacin, 2.5% (25 mg/ml) diluted with sterile saline to a final concentration of 6–8 mg/ml administered in a 0.1 ml infusion volume) was used to prevent infection. Propofol (Methohexital 10 mg/ml @ 0.1 ml infusion) was used to check catheter patency. At this dose propofol administered intravenously causes rats to lose muscle tone and become lethargic within a few seconds of the infusion. Rats that did not display signs of a functional catheter were excluded from further analyses.

#### 2.3.3. Amphetamine self-administration

Following post-surgical recovery, all animals began 14, 2 h fixed-ratio 1 (FR1) amphetamine self-administration sessions. During FR1 self-administration, active lever pressing resulted in a 0.1 mg/kg infusion of amphetamine administered over ~5.9 s^1^ and the concomitant illumination of the cue-light located above the active lever. Illumination of the cue-light terminated at the completion of the amphetamine infusion. Following the infusion and cue-light presentation, the houselight illuminated signaling a 20 s timeout period during which active lever presses were recorded but were inconsequential. Similarly, inactive lever presses were recorded but had no programmed consequence. Catheter patency was re-checked before the first and following the last self-administration session via propofol infusion (10 mg/ml; 0.1–0.15 ml, i.v.). To achieve stable responding, animals were required to average 10 or more amphetamine infusions across all 14 self-administration sessions, as well as maintaining a 2:1 ratio of active to inactive-lever responses over the last 7 self-administration sessions [28, 29]. There were no stability criteria for saline self-administering animals. Data from animals in the amphetamine group with non-patent catheters or from animals that failed to meet the above stability criteria were excluded from all analyses. Five animals were excluded due to a loss of catheter patency and one animal was excluded due to failure to meet the above stability criteria.

#### 2.3.4. Incubation and cue-induced relapse testing

Upon completion of 14 self-administration sessions, all animals entered a forced abstinence period. During the abstinence period all animals remained in their home cages and were not exposed to the drug, self-administration chamber, or drug-associated cues and received daily intraperitoneal injections of either NAC (100 mg/kg dissolved in sterile saline) or the saline vehicle. Cue-induced craving was assessed at the cessation of the abstinence period. Animals received one final injection of NAC (100 mg/kg) or the saline vehicle two hours prior to cue-induced relapse testing. Both the dose of NAC, and the timing of administration during abstinence, are consistent with methods used by Reichel et al. [20], where the authors showed that this dosage was effective in lower cue-induced relapse to cocaine. During the cue-induced relapse test, the motivational impact of drug-associated cues was evaluated by reinforcing active lever presses with presentations of the previously associated drug-cue but no delivery of the drug itself. All relapse tests were 2 h in duration and active lever presses resulted in the illumination of cue lights and houselight. Inactive lever presses were recorded but had no programmed consequence. Given that no amphetamine was available during relapse testing, no timeout period was present during the relapse test. One subset of animals underwent cue-induced relapse testing after one day of withdrawal (WD1), thus allowing us to examine the effects of drug-regulated cues following an acute abstinence period. The other subset of animals underwent cue-induced relapse testing after 14 days of withdrawal (WD14), likewise allowing us to examine the motivational impact of drug-related cues following protracted drug abstinence. Animals tested on WD1 received one injection of NAC (100 mg/kg; ip) two hours to undergoing relapse testing on WD1. Animals tested on WD14 received 14 injections during the abstinence period (one injection per day at a dosage of 100 mg/kg; ip). On WD14, animals received one final injection of either NAC or the saline vehicle two hours before relapse testing. Experimental timelines for animals tested on WD1 and WD14 are shown in Fig. 1A and 1B.

#### 2.3.5. Extinction and cue-induced reinstatement testing

To evaluate whether the efficacy of NAC was contingent on the presence of extinction training prior to relapse testing (as suggested by [20]), a second experiment using an extinction-reinstatement model of drug craving was used. These animals were subjected to the identical self-administration procedures detailed in Section 2.3c. All animals were reliably responding for amphetamine following 14, 2 h long self-administration sessions. Following self-administration, animals daily extinction training where active and inactive lever pressing had no programmed consequence. All sessions were 2 h in duration. Animals were injected with either NAC (100 mg/kg; ip) or the saline vehicle 2 h prior to each of the extinction sessions. On WD14, animals received one final injection NAC or vehicle 2 h prior to the cue-induced reinstatement test in which active-lever pressing resulted in the presentation of the previously associated drug-cue. The cue-induced reinstatement test was 2 h in duration. An experimental timeline for animals undergoing extinction-reinstatement testing can be referenced in Fig. 1C.

#### 2.3.6. Perfusions and immunofluorescence

Immediately following completion of the cue-induced relapse testing, all animals were humanely euthanized via sodium pentobarbitol (Sleep Away) overdose. Animals were transcardially perfused with 0.9% saline and 4% paraformaldehyde and brains were extracted for histochemical analysis. Following extraction, all specimens were transferred to sterilized scintillation vials containing 4% paraformaldehyde for a 4 h post-fixation period. Following sufficient dehydration in 20% sucrose, all samples were snap-frozen on dry ice and transferred to a constant storage temperature of −80°C until cryosectioning. We collected 40 μm sections of the medial prefrontal cortex (mPFC) and nucleus accumbens (ACb).

Immunoassays began by permeabilizing floating sections with 6, 5 min rinses in PBS containing 0.2% Triton-X 100 (1X PBS-TX). Following permeabilization, free-floating sections were blocked in PBS containing 0.2% Triton-X 100 and 5% normal goat serum (Vector Laboratories: S-1000) for 30 min at room temperature. After blocking, sections were again subjected to 3, 5 min rinses in PBS-TX. Free-floating sections were then simultaneously incubated in diluted unconjugated primary antibodies to assess astrocytic densities and astroglial glutamate transporter expression in the mPFC and ACb. GFAP primary antibody dilutions were prepared using a 1:2500 dilution^2^ and xCT primary antibody were prepared using a 1:200 dilution. Both primary and secondary antibodies were diluted in PBS containing 0.2% Triton-X 100. Following primary antibody incubation, sections were again subjected to 3, 5 min rinses in PBS. Tissue was then incubated in fluorescently conjugated secondary antibodies at room temperature for 3 h. Fluorescent-labeling of GFAP+ cells in the mPFC and ACb was assessed using Alexa Fluor 647 goat anti-chicken (1:500 dilution) fluorescent-conjugated secondary antibody. Expression and localization of xCT was assessed using Alexa Fluor 488 goat anti-rabbit fluorescent-conjugated secondary antibody (1:500 dilution). Following three hours of simultaneous incubation in both secondary antibodies, sections were again washed three times with 1X PBS. Sections were counterstained with the DAPI (dihydrochloride; Invitrogen D1306) using a 1:30,000 dilution for 10 min at room temperature. Following cessation of counterstaining, tissue was rinsed three final times in PBS. Free-floating sections were transferred to microscope slides and coverslipped using VectaShield coverslipping medium and coverslips were sealed using standard clear nail polish. Table 1 summarizes the dilutions and sources of each primary and secondary antibody used in this simulatenous fluorescent procedure.

Images were acquired on an Olympus BX63F fluorescence microscope located in the Behavioral Neuroscience core within the Department of Psychological Sciences at Kansas State University. Images were taken at anterior, middle, and posterior regions of each area of interest for all samples. All images were taken 20X magnification (436,000 μm^2^). Given that there were no significant differences in expression across the anterior-posterior axis of each region of interest, mean cell counts for each region were then derived by averaging cell counts across the three images. These aggregated cell counts were used in all immunofluorescence analyses. All image analysis was conducted in Image J. Astrocyte numbers were generated via hand counting of cells showing positive expression of both GFAP and DAPI. Expression of xCT was determined via densitometric analysis of the relative optical density after performing a background correction. This background correction allowed for us to control for any potential differences in background due to autofluorescence that can occur with GFP (wavelength of 488 nm) fluorescence channels. Given that there were no significant differences in xCT optical density across the anterior-posterior axis, the optical density values obtained from densitometric analyses were aggregated into one value for each subject. All image quantification was conducted by individuals blind to experimental conditions.

### 2.4. Data analysis

Self-administration sessions were analyzed using linear multilevel modeling. The number of active lever presses, excluding those that occurred during the 20 s timeout period, served as the primary dependent variable for all analyses. Self-administration multilevel models specified the following fixed effects: the drug that was self-administered (amphetamine or saline), session (a continuous variable consisting of 14 self-administration sessions), and a drug x session interaction, while the intercept and/or session slope of each subject was allowed to vary as random effects. Models utilizing different random effect structures (e.g. intercept or intercept and slope) were compared using Akaike’s Information Criterion (AIC) to determine the optimal model. Active responses that fell three standard deviations away from the grand mean were deemed outliers and were thus excluded from analysis for that session. Additional analyses comparing responding of individual cohorts were also conducted using multilevel modeling. These analyses specified the animal cohort (four level categorical variable) as a fixed effect, and specified both the intercept and session slope of each subject as random effects.

Cue-induced relapse responding was analyzed in animals that previously self-administered amphetamine using a two-way ANOVA. The overall number of active-lever responses served as the primary dependent variable of analysis. Cue-test ANOVAs included the main effects of Treatment (NAC or VEH), Incubation Length (WD1 or WD14), and a Treatment x Incubation interaction term to assess whether the efficacy of NAC treatment is moderated by abstinence duration. Cue-induced reinstatement test sessions were analyzed using an independent- samples *t*-test.

All immunofluorescent data were analyzed using three-way full factorial ANOVAs which included the main effects of Treatment (NAC, VEH), self-administration condition (amphetamine, saline), Incubation Length (Short, Long), and all possible interactions. Given our hypotheses, post-hoc planned comparisons were used where appropriate. Significance levels were set to α = 0.05 for all analyses conducted.

## 3. Theory and calculation

Overall, we predicted that cue-induced active-lever responding would increase at Withdrawal Day 14 when compared to Withdrawal Day 1 for animals that self-administered amphetamine, characteristic of incubation of craving effects. Though incubation effects occur more consistently following extended-access self-administration [4–6], it was predicted that the high dose of amphetamine used here (0.1 mg/kg per infusion) would be sufficient to increase cue-induced craving at Withdrawal Day 14 (WD14) compared to WD1. However, it was predicted that NAC treatment (100 mg/kg; ip) would attenuate this incubation from WD1 to WD14 and would attenuate relapse compared to the saline vehicle at WD1 and WD14. Such findings would be consistent with the findings of Reichel et al. [20] in which NAC (100 mg/kg; ip) was able to attenuate cue-induced relapse to cocaine following 14 days of forced drug abstinence. In Experiment 2, we evaluated whether the efficacy of NAC is contingent on the presence of extinction training, as previous work has suggested [16,20]. Consistent with the work from Reichel and colleagues [20], we predicted that NAC treatment during extinction training would attenuate cue-induced reinstatement to amphetamine-seeking when tested on WD14.

We hypothesized that the mechanism of action that underscores NAC’s efficacy is due to alterations in xCT expression. We predicted that xCT expression would significantly decrease across the duration of drug abstinence for amphetamine animals treated with the saline-vehicle while xCT expression would remain unaffected in animals treated with NAC. Given the impact that cocaine has on impairing glutamate homeostasis [11–13], we predicted that previous exposure to amphetamine during self-administration would decrease xCT expression when compared to saline self-administering animals.

While we hypothesized that the efficacy of NAC would not be related to GFAP+ cell numbers, we predicted that GFAP+ cell quantities will be significantly higher in animals exposed to amphetamine during self-administration. Such findings would be consistent with literature suggesting an increase in GFAP+ cell quantity following exposure to psychostimulants [30].

## 4. Results

### 4.1. Active-responding during 2 h self-administration

Linear multilevel modeling was used to examine differences in active-lever responding across the 14, 2 h self-administration sessions. Analysis of models consisting of two different random effect structures indicated that optimal model performance occurred when both the intercept and subject slope for each session were specified as random effects. Specifying both the intercept and subject slope of each session as random effects resulted in approximately a 50-unit decrease in AIC from a model which solely specified the intercept as a random effect. Importantly, preliminary analysis of active-lever responding indicated that there was no significant difference in average amphetamine- responding between NAC- or VEH-treated rats or animals tested under acute or protracted withdrawal (see Supplemental Fig. 1).

**Fig. 2.**
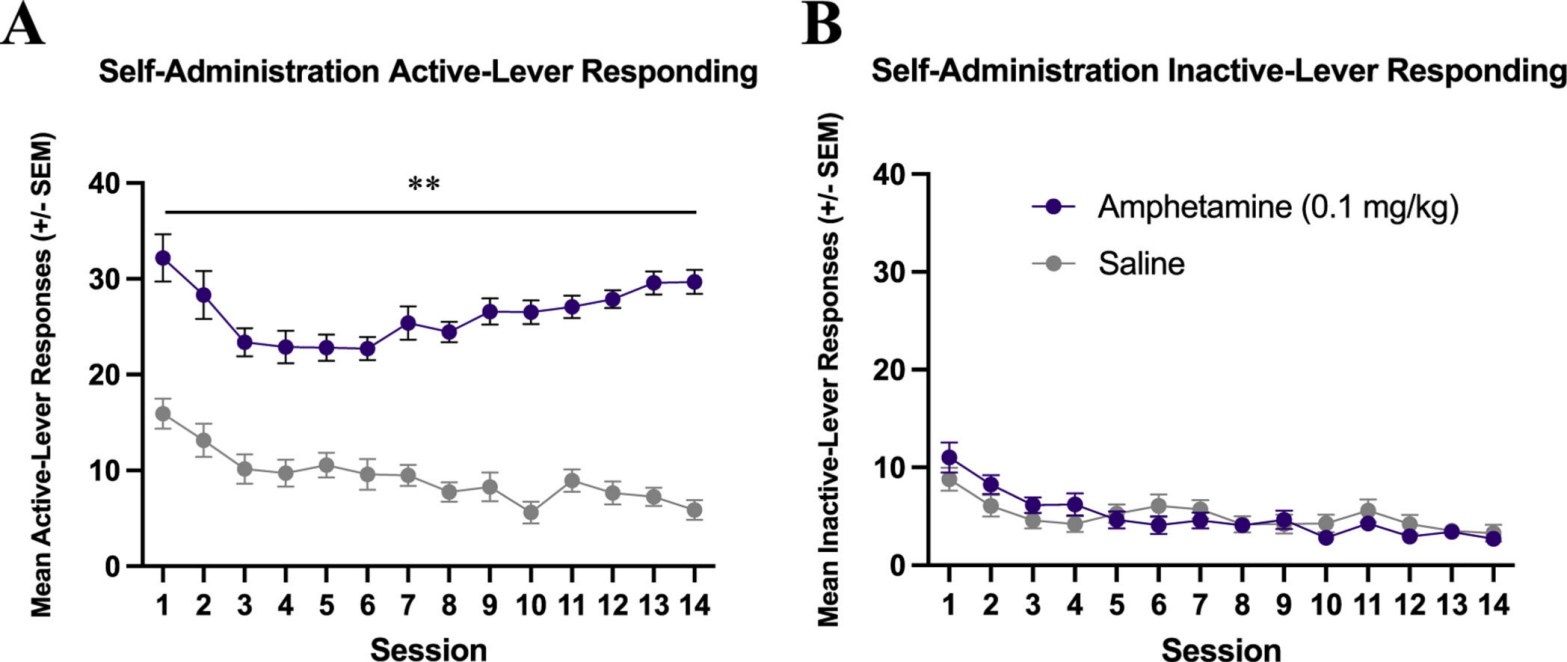
(A) Mean active-lever responding (+/− SEM) for amphetamine (0.1 mg/kg per infusion) and saline during 2 h FR-1 self-administration sessions. The bar and asterisks indicate that active-lever responding was significantly higher for amphetamine when compared to saline, ** *p* < .001. (B) Mean (+/− SEM) inactive-lever responding during 2 h FR-1 self-administration sessions.

Results of the multilevel model revealed a significant fixed effect of drug self-administration condition, *F*(1, 75) = 122.21, *p* < .001, such that active-lever responding for amphetamine was significantly higher than active-lever responding for the saline vehicle (See Fig. 2A). Additionally, results indicate a significant fixed effect of self-administration session, *F*(1, 73) = 6.24, *p* = .01, such that active-lever responding tended to decrease over the course of the 14 self-administration sessions, when collapsed across the drug self-administration condition. More importantly, the drug (amphetamine or saline) x session interaction was a significant predictor of overall active-lever responding during self- administration training,

*F*(1, 73) = 21.66, *p* < .001. Analysis of this interaction revealed that active-lever responding for amphetamine remained constant over the course of self-administration while the slope of the active-lever responding for saline decreased over the course of self-administration. As such, it seemed that the significant fixed effect of session was largely driven by decreasing self-administration in saline self- administering rats. All model fit statistics and parameter estimates are presented in Table 1. Inactive-lever responding during 2 h FR-1 self- administration is shown in Fig. 2B.

### 4.2. Experiment 1: cue-induced relapse following forced abstinence

**Fig. 3.**
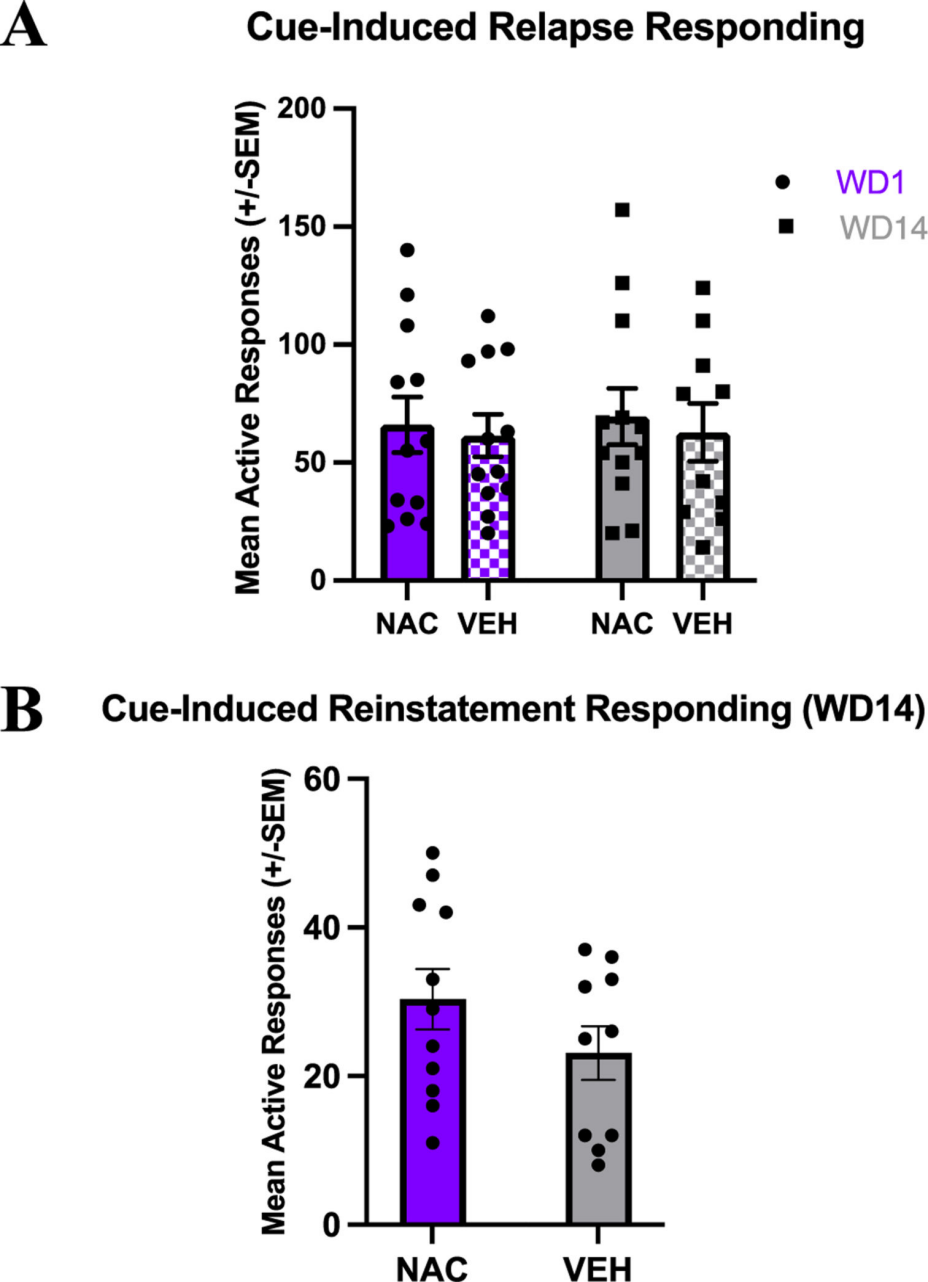
(A) Mean (± SEM) cue-induced active lever responding during the cue- induced relapse test. Purple bars indicate animals tested on WD1 where gray bars indicate animals tested on WD14. Analyses did not indicate any significant effects of the withdrawal period or the treatment condition on cue-induced active lever responding. (B) Mean (+/− SEM) cue-induced active lever responding during cue-induced reinstatement testing on WD14. Analyses indicate that NAC treatment during extinction training was ineffective in lowering cue-induced reinstatement at WD14.

The two-way ANOVA for active-lever responding during the cue- induced relapse test did not indicate that the Incubation Length (WD1 or WD14), Treatment (NAC or VEH), or their interaction were significant predictors of active-lever responding during cue-induced relapse testing. Average cue-induced active-lever responding is shown in Fig. 3A.

### 4.3. Experiment 2: cue-induced reinstatement following extinction

In Experiment 2, we assess cue-induced reinstatement on WD14 following daily extinction training. Responding during the 13 extinction training sessions is presented in Supplemental Fig. 2. The independent samples *t*-test indicated that NAC treatment was ineffective in lowering cue-induced reinstatement at WD14 when compared to the vehicle treatment. Average cue-induced active-lever responding is shown in Fig. 3B.

### 4.4. Immunofluorescence of GFAP within the nucleus accumbens

**Fig. 4.**
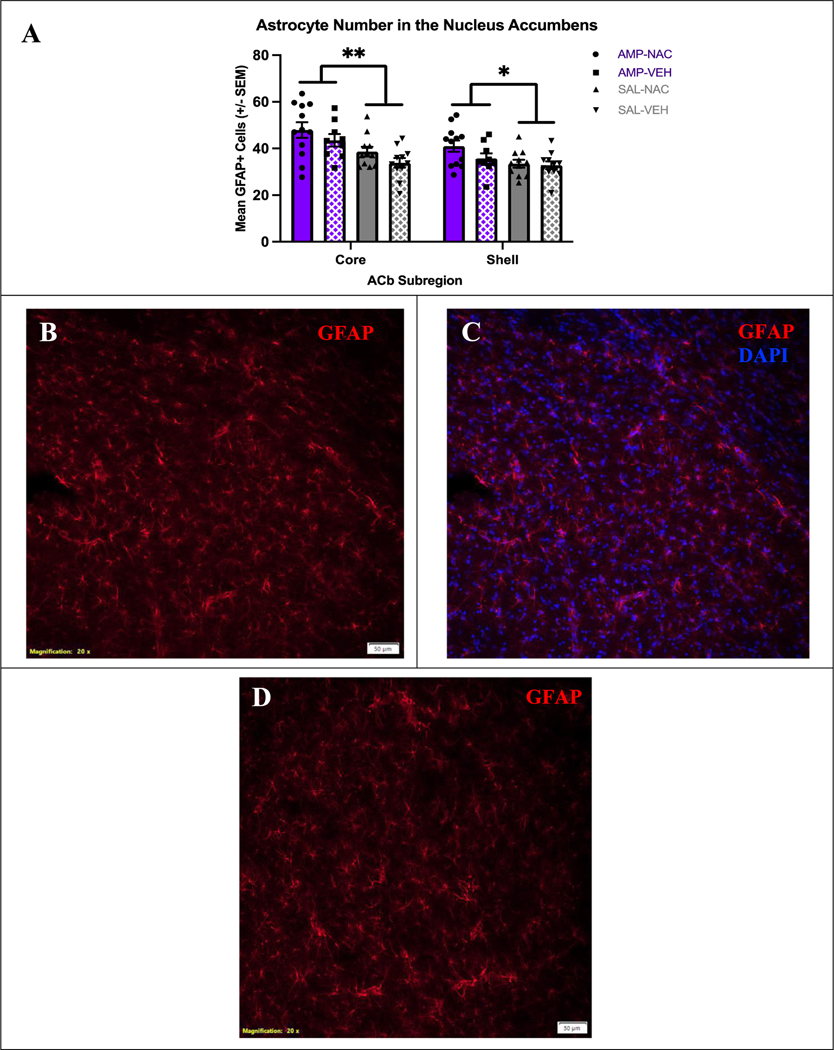
(A) Mean GFAP+ cell expression (+/− SEM) in ACb core and shell. In both subregions, amphetamine increased the quantity of GFAP+ cells. Bars and asterisks indicate a significant difference in GFAP+ cell expression. ** indicates that *p* < .01, * indicates that *p* < .05. (B) Representative 20X image of astrocytes, as indicated by GFAP+ cells, in the ACb core (Total image area of 436,000 μm^2^). (C) Representative 20X image showing GFAP and DAPI colocalization. (D) Representative 20X image of astrocytes, indicated by GFAP+ cells, in the ACb shell (Total image area of 436,000 μm^2^).

Two separate three-way ANOVAs were conducted to assess GFAP+cell quantity within the ACb core and shell. Within the ACb core, results indicated a significant main effect of drug self-administration condition (amphetamine or saline), such that higher GFAP+ cell numbers were seen in animals with a history of amphetamine self-administration, *F*(1, 36) = 11.82, *p* = .002. Similarly, amphetamine self-administration was shown to increase GFAP+ cell numbers in the ACb shell, *F*(1, 36) = 7.04, *p* = .01. These effects are shown in Fig. 4A. Representative 20X images from the ACb core and shell can be seen in Fig. 4B and 4C.

### 4.5. Immunofluorescence of GFAP within the medial prefrontal cortex

**Fig. 5.**
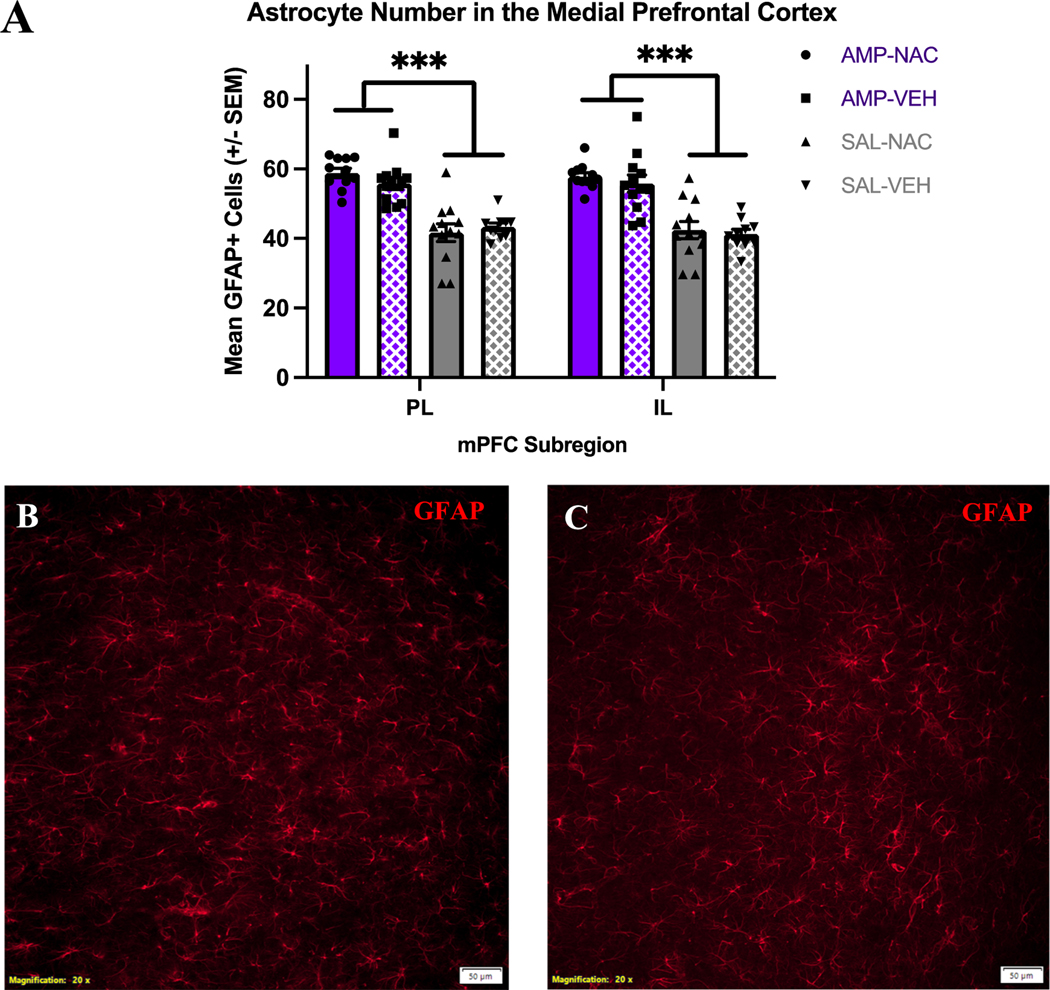
(A) Mean GFAP+ cell expression (+/− SEM) in PL and IL of the mPFC. In both subregions, amphetamine increased the number of GFAP+ cells. Bars and asterisks indicate a significant difference in GFAP+ cell expression. *** *p* < .001. (B) Representative 20X image of astrocytes, as indicated by GFAP+ cells, in the PL (Total image area of 436,000 μm^2^). (C) Representative 20X image of astrocytes, indicated by GFAP+ cells, in the IL (Total image area of 436,000 μm^2^).

Two separate three-way ANOVAs were conducted to determine GFAP+ cell quantity within the prelimbic (PL) and infralimbic (IL) cortices of the medial prefrontal cortex (mPFC). Within the PL, results showed a significant main effect of drug self-administration condition, such that amphetamine self-administering animals showed increased GFAP+ cells, *F*(1, 37) = 75.72, *p* < .001. A similar effect was shown in IL, within amphetamine increasing GFAP+ cell numbers when compared to saline self-administration, *F*(1, 37) = 56.55, *p* < .001. Analysis did not indicate any other significant main effects or interactions within the PL or IL. These effects are shown in Fig. 5A. Representative 20X images from the PL and IL cortices can be seen in Fig. 5B and 5C, respectively.

### 4.6. xCT expression within the nucleus accumbens

**Fig. 6.**
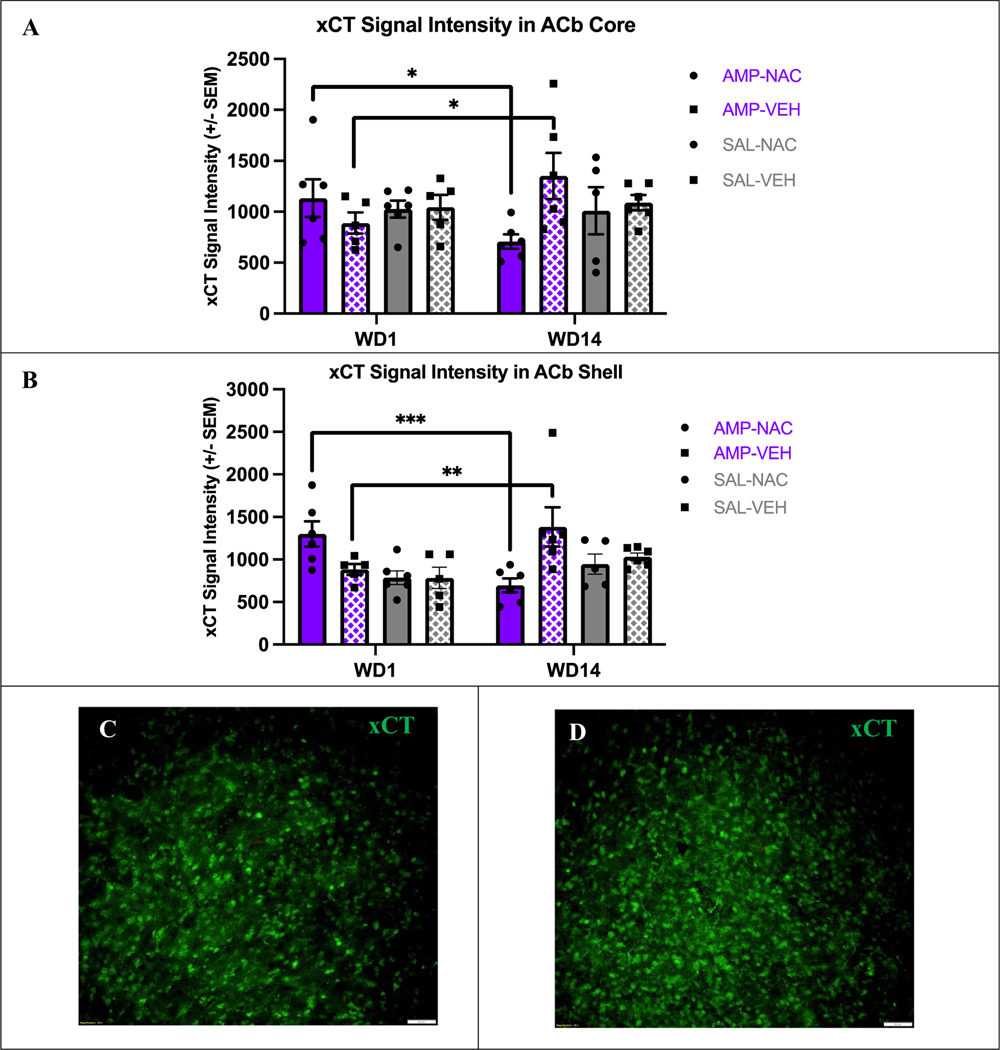
(A) Mean xCT intensity (+/− SEM) in ACb core. Bars and asterisks indicate a significant difference in xCT signal intensity. * *p* < .05, ** *p* < .01, *** *p* < .001. (B) Mean xCT intensity (+/− SEM) in the ACb shell. (C) Representative 20X image (image area = 436,000 μm^2^) of xCT signal intensity in the ACb core. (D) Representative 20X image (image area = 436,000 μm^2^) xCT signal intensity in the ACb shell.

Two separate three-way ANOVAs were used to analyze xCT intensity within the ACb core and shell. Within the ACb core, there was a significant two-way interaction between Incubation length (WD1 or WD14), Treatment (NAC or VEH), *F*(1, 37) = 4.97, *p* = .03. Similarly, post-hoc analyses indicated that repeated NAC treatment decreased xCT expression at WD14 when compared to WD1, *t*(1) = − 2.1, *p* = .04 . Additionally, results indicated a marginally significant three-way interaction between Incubation Length (WD1 or WD14), Treatment (NAC or VEH), and Drug Self-Administration condition (Amphetamine or Saline), *F*(1, 37) = 3.78, *p* = .06 (Fig. 6A; representative image 6C).

Within the ACb shell, the same three-way interaction was found to be significant, *F*(1, 37) = 7.88, *p* = .008. Post-hoc planned comparisons of animals that previously self-administered amphetamine indicated that repeated treatment with NAC decreased xCT expression from WD1 to WD14, *t*(1) = − 3.46, *p* = .001 (Fig. 6B; representative image 6D).

Interestingly, the two-way interaction between Treatment and Self- Administration condition was not found to be statistically significant in either the ACb core or shell. Despite the fact that this two-way interaction did not reach the *a priori* significance level, post-hoc probing was conducted to examine differences in xCT intensity for amphetamine and saline self-administering animals that received the vehicle treatment. Results of this analysis found no significant difference in xCT expression between amphetamine-exposed and amphetamine-naïve animals in either subregion of the ACb.

### 4.7. xCT expression within the medial prefrontal cortex

**Fig. 7.**
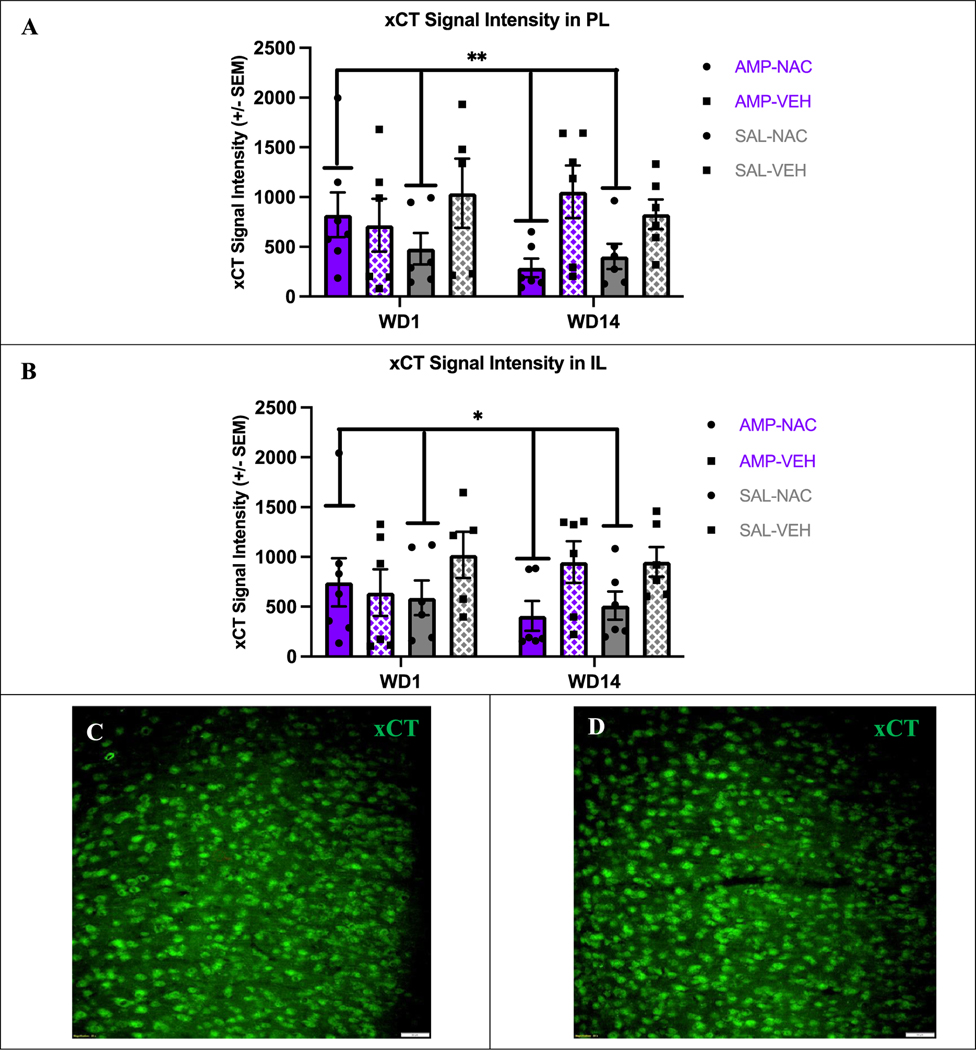
(A) Mean xCT intensity (+/− SEM) in PL. Bars and asterisks indicate that NAC treatment significantly lowered overall xCT signal intensity compared to VEH-treated animals, *p* < .05, ** *p* < .01. (B) Mean xCT intensity (+/− SEM) in IL. (C) Representative 20X image (image area = 436,000 μm^2^) of xCT signal intensity in PL. (D) Representative 20X image (image area = 436,000 μm^2^) of xCT signal intensity in IL.

Two separate three-way ANOVAs were used to analyze xCT intensity within the PL and IL cortices. Within the PL, there was a significant main effect of Treatment (NAC or VEH), such that NAC-treated animals showed lower xCT expression, when collapsed across other experimental conditions, *F*(1, 40) = 7.33, *p* = .01. This effect is shown in Fig. 7A. No other main effects or interactions were found to be significant.

Similarly, the main effect of treatment (NAC or VEH) was shown to be lone significant effect within the IL. Again, NAC treatment was shown to significantly decrease overall xCT intensity when compared to VEH treated controls, *F*(1, 40) = 5.44, *p* = .02 . This effect is shown in Fig. 7B. Fig. 7C and 7D show representative 20X micrographs of xCT expression within the PL and IL, respectively.

Again, our analyses did not indicate that there was a significant two-way interaction between the Treatment and Self-Administration conditions. However, post-hoc probing was still conducted to examine potential differences in xCT expression imposed by amphetamine self- administration in animals that received the vehicle treatment. As in the ACb, our results did not indicate any significant in xCT expression between amphetamine-exposed or amphetamine-naïve animals in either the PL or IL. The significance of these effects in the mPFC, and those in the ACb, will be explored in greater detail in the discussion.

## 5. Discussion

### 5.1. Summary of main results

The current study offers several key insights which help to fill voids in the understanding of the treatment and neurobiology of cue-induced relapse to amphetamine. First, our data show that incubation of drug craving did not occur following short-access amphetamine self-administration. Second, the therapeutic NAC is not effective in attenuating cue-induced relapse during acute or protracted abstinence from amphetamine-use. Additionally, our results demonstrate that NAC treatment during extinction training is not effective in lowering cue- induced reinstatement when assessed at Withdrawal Day 14. Third, these data show that amphetamine significantly increases astrocyte expression within both regions of the mPFC and ACb. Finally, these data suggest that repeated NAC treatment during the abstinence period can actually decrease xCT expression within both the mPFC and ACb. Together, these findings suggest that while NAC decreases xCT expression, it has limited effectiveness for the attenuation of cue-induced craving for amphetamine.

### 5.2. Incubated cue-induced craving does not occur following 2 h continuous access self-administration sessions for amphetamine

In the current study, it was predicted that the high dose of amphetamine utilized would be sufficient to produce incubation in drug craving from Withdrawal Day 1 to Withdrawal Day 14, despite only being available in short-access self-administration sessions. However, animals tested on Withdrawal Day 14 did not show higher levels of cue-induced craving when compared to those tested at Withdrawal Day 1 as predicted. This finding merits several points of discussion as it highlights potential voids in previous literature on the incubation of cue-induced craving for amphetamine.

There is a plethora of literature showing a reliable and robust increase in cue-induced craving following forced drug-abstinence across several different drugs of abuse. For instance, there is evidence illustrating incubation of craving for methamphetamine [31], cocaine [4, 32], heroin [33,34], nicotine [35], and alcohol [36]. However, a review of this literature suggests that the most robust incubation effects, regardless of drug or drug class, occur following extended-access self-administration models (i.e. 6–9 h of self-administration per session) [6,32]. While short-access self-administration models (1 or 2 h of self--administration per session) can elicit increases in cue-induced drug-seeking during abstinence, these effects occur rather inconsistently and seem to be most prominent following short-access to cocaine [37,38].

Based on current understanding, incubation of cue-induced drug-craving has not be observed for amphetamine. One plausible conclusion for why incubation in cue-induced craving was not observed in the current study is that the timeline for the incubation of amphetamine craving requires a different abstinence period in order to elicit the effect. Increases in drug-seeking for cocaine, heroin, nicotine, and methamphetamine are evident within a manner of weeks of abstinence to the drug [31,33,35,39]. As such, it is likely that long-access self--administration procedures are required to observe incubations in cue-induced amphetamine craving. Recent work from our lab using short-access amphetamine self-administration supports this conclusion. Previously, we did not see an increase in craving at Withdrawal Day 40 when compared to Withdrawal Day 1 [27], thus suggesting that the incubation of amphetamine craving is likely reliant on long-access self-administration. Evidence from both cocaine and methamphetamine self-administration supports this conclusion given that long, but not short-access, self-administration procedures result in accumulations of CP-AMPA receptors at excitatory synapses in the ACb core [40,41].

### 5.3. N-acetylcysteine (NAC) does not attenuate cue-induced relapse or reinstatement to amphetamine during acute or protracted withdrawal

The current study was designed to extend the existing literature showing NAC’s efficacy to reduce relapse to amphetamine-associated cues. The methods utilized were consistent with work showing that NAC was effective in lowering cue induced drug-seeking for both methamphetamine and cocaine [20,21]. Given its efficacy for treating relapse to cocaine, we derived both our withdrawal period (14 days of abstinence to the drug) and NAC dose (100 mg/kg; ip) from studies showing NAC’s potential efficacy in attenuating cue-induced relapse to cocaine [20] and reinstatement to methamphetamine [21]. While both studies found that NAC (100 mg/kg; ip) was effective for lowering cue-induced cocaine relapse or methamphetamine reinstatement, this same dose is insufficient to lower cue-induced relapse to amphetamine.

It is important to note that the preponderance of studies showing NAC’s efficacy in mitigating cue-induced drug-seeking across multiple drugs of abuse did not assess cue-induced craving at more than one relapse or reinstatement session during the abstinence period [20,21]. As such, we are confident that the efficacy of NAC is not contingent on incubations in craving during abstinence. Therefore, the lack of such an incubation effect in the current study does not present a reason why NAC failed to attenuate cue-induced drug craving at either WD 1 or WD 14.

The finding that NAC was ineffective at lowering cue-induced relapse during either acute or protracted withdrawal conditions is noteworthy and highlights important distinctions between therapeutic interventions for different drugs of abuse. Though NAC has been shown to be effective in lowering cue-induced relapse to cocaine [16,19,20], the current study has shown that this efficacy does not extend to amphetamine. Additionally, the efficacy of NAC in mitigating cue-induced craving for methamphetamine has been inconsistently demonstrated in both preclinical and clinical models. For instance, while Siemsen et al. [21] found that NAC was effective in lowering cue-induced reinstatement to methamphetamine, work from Charntikov et al. [42] demonstrated that NAC was not able to attenuate cue-induced reinstatement to methamphetamine. This inconsistency is also evident in clinical human studies with one study showing a reduction in methamphetamine cravings following NAC treatment [43], but a comparable study showing no reduction in methamphetamine craving following NAC treatment [44]. Overall, this review of the literature suggests that the efficacy of NAC treatment for methamphetamine is likely inconsistent, at best. Taken together, these findings suggest that NAC’s efficacy may be limited to certain psychostimulants (e.g. cocaine) but does not extend to other compounds within the same drug class (e.g. amphetamine and methamphetamine).

Though cocaine, methamphetamine, and amphetamine are all classified as psychostimulants, there are well-defined neurochemical distinctions and mechanisms of action. The primary mechanism mediating the abusive potential of psychostimulants is their ability to bind dopamine transporters (DAT) [45,46]. Amphetamine and cocaine differ in the mechanism by which dopamine is eliminated and trafficked from the synaptic, and extrasynaptic areas [47–49]. Amphetamine and methamphetamine differ in their impacts on glutamate release within the ACb and mPFC. Shoblock et al. (2003) showed that amphetamine and methamphetamine evoke opposite glutamatergic changes in the ACb and mPFC [50]. Specifically, amphetamine evoked much larger increases in extracellular glutamate in ACb than those evoked by methamphetamine. This effect was reversed in the mPFC, with methamphetamine evoking larger increases in extracellular glutamate when compared to amphetamine. Therefore, it is entirely possible that the differences in these mechanisms could result in differences in the efficacy of NAC for different psychostimulants.

Another possible explanation for the discrepancies in the efficacy of NAC could be the presence or absence of extinction training following self-administration. This point has been made in previous literature using NAC as a relapse preventative, citing that the effectiveness of NAC is increased in the context of extinction training, conceivably due to its potential cognitive-enhancing effects [20]. To address whether the efficacy of NAC is contingent on the presence of extinction training, we conducted Experiment 2 in which animals underwent daily extinction training following NAC supplementation. Our results from Experiment 2 showed that NAC was ineffective in attenuating cue-induced reinstatement when compared to vehicle-treated subjects at WD14. These results bolster the conclusion that the efficacy of NAC in reducing cue-induced craving likely varies across different psychostimulants. However, it is important to note that given the neuroadaptations that accompany extinction learning [51], it is important for future research exploring NAC, and other relapse pharmacotherapies, to explore whether efficacy changes across drug-class and the presence or absence of explicit extinction training.

### 5.4. Astrocyte expression within the nucleus accumbens and medial prefrontal cortex is altered by amphetamine self-administration

The current study found that previous exposure to amphetamine during self-administration was associated with increases in the number of GFAP+ astrocytes within the ACb core and shell as well as both the prelimbic (PL) and infralimbic (IL) cortices of the medial prefrontal cortex (mPFC). This finding is in contrast to work showing that methamphetamine exposure did not alter astrocytes in the ACb [21]. However, these results are in keeping with previous literature showing that exposure to cocaine can increase astrocyte expression [30]. One potential explanation for why such an increase would occur is that this could serve as a compensatory mechanism for declining xCT expression on the surface of astrocytes. However, this increase could prove to be deleterious due to potential neuroinflammation that would occur with large increases in astrocyte expression. Neuroinflammation has been hypothesized to contribute to drug-induced neurotoxicity for a number of drugs of abuse [52]. Another alternative hypothesis for why astrocyte expression would increase following exposure to amphetamine is due to a potential increase in extracellular glutamate in response to amphetamine. These increases in glutamate may stimulate mGluR5 receptors, which are abundant on the surface of astrocytes. Activation of mGluR5 has been shown to increase astocyte [mehtl-^3^H]thymidine incorporation [53]. The significance of the interaction between psychostimulants and the mGluR5 system is also ameliorated by the finding that mGluR5 gene deletion attenuates the rewarding and psychomotor effects of cocaine in mice [54].

### 5.5. The effect of repeated NAC treatment on xCT expression

The current study found that repeated injections of NAC (100 mg/kg) decreased xCT intensity within the ACb core and shell at WD14 when compared to WD 1 (see Fig. 6). Additionally, within the mPFC, NAC treatment decreased xCT signal intensity, independent of other experimental conditions (see Fig. 7).

Given that xCT-mediated glutamate release is the primary mechanism of action for NAC, the finding that repeated NAC treatment decreased xCT expression, when measured at WD 14, is quite surprising. This finding suggests that the xCT-mGluR2/3 system may not be implicated in amphetamine-use disorder and cue-induced relapse to amphetamine-seeking. This conclusion is supported by the lack of difference in the xCT signal intensity of amphetamine self-administering of amphetamine-naïve animals shown here. Taken together, these results suggest that xCT may not be the mechanism responsible for cue-induced relapse to amphetamine during drug abstinence.

### 5.6. Future directions and conclusions

While we do show that NAC given at a dose of 100 mg/kg (i.p.) was ineffective at lowering cue-induced relapse or reinstatement to amphetamine at either WD1 or WD14, it is worth noting that we tested only one dose of NAC. While this dose of NAC was previously shown to be effective in mitigating cue-induced relapse to cocaine [20] and cue-induced reinstatement to methamphetamine [21], it is possible that a different dose may be required to treat cue-induced craving for amphetamine. Future work assessing the efficacy of NAC in this application should explore different doses to further explore any efficacy that NAC may have for treating cue-induced amphetamine craving.

Given that notably few experiments have looked at the effect of NAC on xCT expression and the fact that this is, to our knowledge, the first time that GFAP and xCT have been assayed together in the same immunofluorescence procedure following psychostimulant exposure, we elected to limit our initial analysis to male subjects. However, preclinical and clinical evidence suggests females may be more suscueptible to cue-induced craving and relapse when compared to males [55–57]. In light of the number of documented sex differences in cue-induced craving and relapse, future research in this area should further evaluate the effects detailed here in female subjects.

The results of the current study suggest that NAC is likely ineffective for attenuating cue-induced craving for amphetamine, despite previous demonstrations of its potential for attenuating cue-induced relapse to cocaine [20] and cue-induced reinstatement to methamphetamine [21]. More broadly, these findings suggest that treatments and therapeutics used to treat cue-induced craving during drug abstinence cannot take a one-size fits-all approach, potentially even for treating drugs of abuse within the same drug class (e.g. cocaine and amphetamine). Rather, it is critical that research continues to discover, develop, and refine treatments specific to amphetamine-use disorder.

## Supplementary Material

1

2

3

## Figures and Tables

**Table 1:** Fixed effects and model fit statistics for self-administration multilevel model using both intercept and subject slope of each session as random effects.

					Model Statistics		
	*B*	*SE*	*t*	*p*	$R^{2}$	$R_{adjusted}^{2}$	*AIC*

Intercept	20.39	1.08	18.90	<0.001	0.74	0.74	7324.5
Session	− 0.25	0.10	−2.50	.01			
Drug (AMP/SAL)	8.38	0.76	11.50	<0.001			
Drug * Session	0.47	0.10	4.65	<0.001			

Note: Significance is indicated by boldfaced font.

## Data Availability

Data will be made available on request.

## Footnotes

1 The infusion speed is adjusted for body weight, but the average infusion speed is approximately 5.9 s.

2 This antibody dilution was informed by prior GFAP IHC-enzymatic detection (data unpublished)
