# Supplementary material for: Inefficacy of N-acetylcysteine in mitigating cue-induced amphetamine-seeking
Source: Addict Neurosci. Author manuscript; Available in PMC 2024 Jan 11. (PMC10783794; doi:10.1016/j.addicn.2023.100119)
Supplement: 1 [file NIHMS1926091-supplement-1.docx]

*Supplement Figure 1.* Mean active-lever responding (+/- SEM) for amphetamine during 2-hr FR-1 self-administration. Analysis indicated that there was no significant difference in average amphetamine-intake between NAC- or VEH-treated rats or animals tested under acute or protracted withdrawal.
