# Supplementary material for: Inefficacy of N-acetylcysteine in mitigating cue-induced amphetamine-seeking
Source: Addict Neurosci. Author manuscript; Available in PMC 2024 Jan 11. (PMC10783794; doi:10.1016/j.addicn.2023.100119)
Supplement: 2 [file NIHMS1926091-supplement-2.docx]

*Supplemental Figure 2.* Active-lever responding during extinction training.
