# Supplementary material for: Inefficacy of N-acetylcysteine in mitigating cue-induced amphetamine-seeking
Source: Addict Neurosci. Author manuscript; Available in PMC 2024 Jan 11. (PMC10783794; doi:10.1016/j.addicn.2023.100119)
Supplement: 3 [file NIHMS1926091-supplement-3.docx]

| **Antibody** | **Dilution (either PBS or PBS-TX as diluent)** | **Vendor** | **Product #** | **Validation Technique**  **(From Vendor)** |
| --- | --- | --- | --- | --- |
| Chicken anti-GFAP (polyclonal) | 1:2,500 | Novus Biologicals | NBP1-05198 | IHC , ICC/IF, WB |
| Rabbit anti-xCT (polyclonal) | 1:200 | Novus Biologicals | NB300-318 | IHC, ICC/IF, WB |
| Goat anti-Chicken IgY (H+L), Alexa Fluor 647 | 1:500 | Invitrogen | A32933 |  |
| Goat anti-Rabbit IgG (H+L), Alexa Fluor 488 | 1:500 | Invitrogen | A32731 |  |

Supplemental Table 1. *Summary of reagents used in simultaneous immunofluorescence procedure.*
